# The HLA-Cw6 Dilemma: Is It Really an Outcome Predictor in Psoriasis Patients under Biologic Therapy? A Monocentric Retrospective Analysis

**DOI:** 10.3390/jcm9103140

**Published:** 2020-09-28

**Authors:** Martina Burlando, Roberto Russo, Andrea Clapasson, Luca Carmisciano, Anna Stecca, Emanuele Cozzani, Aurora Parodi

**Affiliations:** 1 Di.S.Sal. Section of Dermatology, University of Genoa, Via Pastore 1, 16132 Genoa, Italy; russoroberto@outlook.com (R.R.); clpndr@yahoo.it (A.C.); emanuele.cozzani@unige.it (E.C.); aurora.parodi@unige.it (A.P.); 2 IRCCS San Martino Polyclinic Hospital, Largo Rosanna Benzi 10, 16132 Genoa, Italy; 3 Di.S.Sal. Section of Biostatistics, University of Genoa, Via Pastore 1, 16132 Genoa, Italy; lucacarmisciano@gmail.com; 4 Euroimmun Italia Medical Diagnostic, Corso Stati Uniti 4, 35100 Padua, Italy; a.stecca@euroimmun.it

**Keywords:** psoriasis, pharmacogenomics, HLA-Cw6, biologic therapy, biologic drugs, PASI90

## Abstract

HLA-Cw6 is one of the most strongly associated psoriasis susceptibility alleles. Data regarding correlation between HLA-Cw6 status and biologic treatment outcomes are divergent. The aim of our study in our cohort of psoriatic patients was to explore if the HLA-Cw6 status influences the response rate to biologic therapies at 16 and 48 weeks. One hundred and one psoriatic patients eligible for biologic therapies were enrolled. HLA-C*06 alleles were detected from their blood samples. The effectiveness of antipsoriatic treatments was reported as 90% Psoriasis Area and Severity Index reduction (PASI90). All biologics showed efficacy at week 16, without significant differences between one another. HLA-Cw6 status did not seem to affect baseline characteristics, or treatment response at week 16. At week 48, IL-12/23 and IL-17 targeting drugs were more effective on Cw6-positive patients than on Cw6-negative patients. Conversely, TNF-targeting drugs seemed to be more effective on Cw6- negative patients than on Cw6-positive patients. The HLA-Cw6 test could well deserve to be integrated into the clinical laboratory work-up supporting the choice of the correct biologic.

## 1. Introduction

Psoriasis is a chronic immune-mediated skin disease [1,2]. Advances in our understanding of the pathogenesis of psoriasis have enabled the development of more selective anti-inflammatory agents known as biologics. These are manufactured antibodies or fusion proteins, which block pro-inflammatory cytokines or their receptors [3]. The response to these agents is heterogeneous [4,5]. Finding the best biologic for the individual patient is a process of trial and error, which may lead to a long period of suboptimal clinical effectiveness and unnecessary costs. As the number of biologics registered for the treatment of psoriasis increases, so does the need for biomarkers to guide personalized therapeutic decisions.

Genetic variants might explain part of the observed heterogeneity and serve as predictors for treatment response; this field of research is known as pharmacogenetics.

HLA-Cw6 is one of the most strongly associated psoriasis susceptibility alleles. It has been repeatedly observed to affect disease course, phenotypic features, severity, comorbidities, and treatment outcomes [6].

Regarding treatment outcomes, taking into consideration the biologic therapies nowadays available, the data are divergent. Gallo et al. found no correlation between HLA-Cw6 status and treatment response to adalimumab, etanercept, or infliximab (anti-TNFα drugs) [7]. Regarding secukinumab (anti-IL17 drug), Costanzo et al. reported that a similar PASI 90 response was achieved irrespective of HLA-Cw6 status at week 16, and mean Psoriasis Area and Severity Index (PASI) was comparable at week 16 and 24 [8]. Data on ustekinumab are divergent. Several studies in fact have demonstrated a significant increase of treatment response in HLA-Cw6 positive patients treated with ustekinumab (anti IL-12 and IL-23 drug) [9,10]. Moreover, the analysis of a phase III trial showed that there was a differential response to ustekinumab in HLA-Cw6 positive versus negative patients; however, this difference is modest, particularly at the higher response rate thresholds and later time points [11]. However, the association between the HLA-Cw6 polymorphism and response to ustekinumab was not observed in a Spanish study [12]. Recently a large study based on the English national psoriasis registry shed light on this dilemma. Cw6-negative patients seemed to respond better to adalimumab than ustekinumab, especially in the presence of psoriatic arthritis, but no data were reported regarding anti IL17 drugs [13].

Therefore, the aim of our study in our cohort of psoriatic patients was to explore if the HLA-Cw6 status influences the response rate to all the biologic therapies available on the market at that time, in both short (16 weeks) and long periods (48 weeks).

## 2. Experimental Section

Our study included 101 patients affected by moderate to severe plaque psoriasis. The number of patients selected was based on the HLA-Cw6 kit availability. Between September and December 2017, the first 101 patients related to our Clinic with indication of biologic therapy were enrolled in the study and followed up until December 2018. Patients with psoriatic arthritis were excluded from the study in order to make our sample as homogeneous as possible.

To choose the correct biologic agent for each patient, we focused on the PASI score and comorbidities. Patients with a severe PASI (more than 15) or with latent tuberculosis were given anti IL-17 (secukinumab or ixekizumab) drug antibodies, known to be the fastest in action among biologics and with a low risk of activating tuberculosis [14]. Younger patients, travelers, or those with needle-phobia were given anti IL12–23 (ustekinumab) every 12 weeks, highly appreciated for its dosing period [15]. Patients with a moderate PASI (10–15) were given an anti TNFα (adalimumab, etanercept, infliximab, certolizumab, golimumab) [14]. As a result, the numbers of patients treated with any particular drug were modest. For this reason, we clustered biologics according to their class of action, making the group size homogeneous.

All the therapies were administered at the labelled posology and all patients were treated out of any clinical trial. Patients were not allowed to use systemic or topical medications except emollients. Patient demographic characteristics and clinical/anamnestic data were collected: HLA-Cw6 status, age, gender, disease duration, baseline PASI score, and presence of comorbidities (i.e., hypertension, obesity, etc). Information about previous systemic and/or biologic antipsoriatic therapies was also collected. The effectiveness of antipsoriatic treatments is reported as 90% PASI reduction (PASI90) at different timepoints (week 16 and week 48).

The present study was conducted in accordance with the Helsinki Declaration of 1975, as revised in 1983, on Ethical Principles for Medical Research Involving Human Subjects. It was approved by our ethical committee with the code: CE2585PRNO240820, 426/2020 10822. All eligible patients provided written informed consent.

For isolation and purification of genomic DNA, 200 μL whole blood sample treated with EDTA QIAamp^®^ DSP DNA Blood Mini Kit (QIAGEN GmbH, Hilden, Germany) was used, following the protocol in according to the manufacturer instruction. Final elution volume was 50 μL.

EUROArray (EUROIMMUN AG, Luebeck, Germany) workflow consisted of a first step including the amplification of target regions, b-globin gene fragment (as endogenous positive control), and several other reaction controls from genomic patient DNA samples by multiplex PCR. The obtained products were labelled with a fluorescence dye. In the second step, the PCR products were analyzed by a microarray-approach. The immobilized probes (20 to 70 nucleotides length) on a slide, complementary to target DNA, were hybridized to PCR products using TITERPLANETM incubation technology, obtaining fluorescing spots. Then, they were detected by a specific Microarray Scanner (EUROIMMUN AG, Luebeck, Germany). The presence of a target allele in DNA samples of patients was detected by a fluorescent signal generated by a specific spot, which was automatically evaluated by EUROArrayScan Software (EUROIMMUN AG, Luebeck, Germany).

For HLA-Cw6 test, a region of the HLA-C*06 gene was amplified. All currently known HLA-C*06 alleles were detected, with the exception of four alleles for which a positive reaction was not 100% ensured (C*06:15, C*06:23, C*06:47 and C*06:49N) and two alleles which were not detected (C*06:30 and C*06:35).

The EUROArray test was performed according to the manufacturer’s protocol. Briefly, a PCR master mix was prepared mixing 10 μL of Mix A and 10 μL of Mix B, then 5 μL of DNA sample was added. PCR was carried out using an Applied Biosystem 2720 Thermal Cycler (ThermoFisher Scientific, Waltham, MA, USA) with the specific thermal protocols: for the HLA-Cw6 test, a step of 5 min at 95 °C to denaturate followed by 10 s at 95 °C, 20 s at 68 °C to anneal and 20 s at 72 °C with extension for 32 cycles, then 3 cycles of 10 s at 95 °C and 5 s at 68 °C followed by a step of 4 °C until use. Each PCR product was mixed with 65 μL of Hybrydization Buff. The mix solution was applied to a corresponding reaction field on TITERPLANETM and incubated on a microarray slide for 1 h at 45 °C. After hybridization, slides were washed and dried prior to analysis using Microarray Scanner and EUROArrayScan Software.

Regarding statistical analysis, continuous variables were summarized with mean and standard deviation (SD) or median and inter quartile range (IQR), discrete variables were summarized as count and percentage. T-test, Mann–Whitney test, and chi-squared test were used as appropriate to detect the association between baseline characteristics and Cw6 status. The 90% reduction from the PASI before treatment was used as the binary outcome (PASI90). The chi-squared test was used to assess homogeneity of PASI90 outcome across drugs. Two separate logistic models were fitted (for 16 and 48 weeks) using PASI90 as dependent variable, CW6 status and treatment as explanatory variables and CW6*treatment as interaction term, Odds Ratio (OR) and their 95% Confidence Interval (95% CI) were reported. Likelihood ratio test was used to compare the nested model and therefore to estimate the improvement in model fit due to the addition of the interaction term. Multivariable models were used to control confounders: naïve status, age, gender, disease duration, and initial PASI score. Analyses were performed with R 3.6.0.

## 3. Results

### 3.1. Baseline Characteristics

Overall, 101 patients were collated; 23 (23%) were Cw6-positive, in line with the literature for Italian patients with psoriasis [16]. No significant associations were observed between age (*p* = 0.28), gender (*p* = 0.93), initial PASI score (*p* = 0.37) or disease duration (0.12) and the treatment. However, the previous biological-naïve status was heterogeneous among treatments (90% of TNF were naïve, 61% of IL-12/23, and 68% of IL-17, *p* = 0.013). No significant associations between Cw6 status and baseline characteristics were detected (Table 1). Cardiovascular diseases were the most reported comorbidities.

### 3.2. Treatment Response

Results are summarized in Table 2.

#### 3.2.1. Week 16

Overall, 95 patients (94%) reached PASI90 and the effect appeared to be homogeneous across drug classes (𝜒^2^ (2) = 1.90, *p* = 0.386). There was a similar proportion of patients achieving PASI90 in Cw6 positive (96%) and negative (94%) groups, thus Cw6 does not appear to be an overall prognostic factor at week 16 (OR = 1.53, 95% CI = 0.22–30.7, *p* = 0.710). All classes of drugs seemed to have similar effects, regardless of the Cw6 status (*p* for interaction = 0.610).

#### 3.2.2. Week 48

Seventy-five patients who achieved PASI90 at week 16 (79%) maintained PASI90, while 20 (21%) did not. There was a similar proportion of patients maintaining PASI90 in the Cw6 positive and negative groups (74% in both), thus Cw6 does not appear to be an overall prognostic factor (OR = 1.09, 95% CI = 0.38–3.52, *p* = 0.874). Drugs targeting IL12/23 and IL17 seemed to be more effective on Cw6-positive patients than on Cw6-negative patients. Conversely, TNF-targeting drugs seemed to be more effective on Cw6-negative patients than on Cw6 positive patients. Hence, Cw6 appears to be a predictor of the treatment effect at week 48 (*p* for interaction = 0.050). Among patients who did not respond at week 16, no one achieved PASI90 at week 48. Adjusting the logistic model for the naïve status did not significatively improve the model fit (Likelihood ratio test *p* = 0.93). As sensitivity analyses the interaction term, contribution was estimated with and without adjustments for the naïve status, age, gender, disease duration, and initial PASI score, returning similar estimates (𝜒2 (2) = 6.42, *p* = 0.040 vs. 𝜒2 (2) = 6.00 *p* = 0.050).

No adverse events were reported in either group for any of the biologics.

## 4. Discussion

The relationship between biologics and HLA-Cw6 has been widely investigated owing to the high cost of biologics [6,17]. Numerous subsequent trials across multiple nations established that HLA-Cw6 status was not indicative of treatment response to adalimumab and etanercept [18], although in a more recent report HLA-Cw6 positivity has been linked to better response to adalimumab at week 24 [19]. Costanzo et al. [20] described similar PASI90 responses at week 16, as well as comparable mean PASI scores at weeks 16 and 24, irrespective of HLA-Cw6 status. Data on ustekinumab are divergent. Talamonti et al. [9,10] reported that patients carrying the HLA-Cw6 allele showed significantly faster and increased response, with 96.3% of patients reaching PASI75 at week 28 vs. 72.7% in the HLA-Cw6-negative group. In addition, a large European cohort study also showed that significantly more patients carrying the HLA-Cw6 allele achieved PASI50 and PASI75 at weeks 4 and 12 [21]. Disproving all of the above, a retrospective study that analyzed phase III trials for ustekinumab found that the differences in response between HLA-Cw6-positive and HLA- Cw6- negative patients at later end points (PASI90 at week 24 and PASI100 at week 28) were only modest [11]. Despite the association between HLA-Cw6 status and response to ustekinumab, other genetic and environmental factors cannot be ignored. Dand et al. published the largest observational study regarding this topic. They focused on adalimumab and ustekinumab. They demonstrated that HLA-C*06:02-negative patients with psoriasis are significantly more likely to respond to adalimumab than to ustekinumab but that there is no significant benefit to adalimumab over ustekinumab in HLA-C*06:02-positive patients [13].

Analyzing our results, all biologics showed efficacy at week 16, without significant differences between them. Twenty patients who were responders at week 16 experienced a response loss at week 48. On the other hand, all patients who did not respond at week 16 also failed to achieve PASI90 at week 48.

HLA-Cw6 status did not seem to affect treatment response at week 16. All Cw6-positive patients treated with anti IL17 or anti IL12/23 who achieved PASI90 at week 16 maintained PASI90 at week 48. Conversely, considering all Cw6-negative patients treated with the drugs mentioned above who achieved PASI90 at week 16, 20% of them failed to conserve PASI90 at week 48. Thus, HLA-Cw6 positivity seemed to predict a long-lasting response to these biologics.

What emerged from our data is that HLA-Cw6 status in patients receiving biologics may help to identify drug responders at week 48 (χ2 (2) = 6.00, *p* = 0.050).

Our study has some limitations: it is based on a relatively modest sample size and different drugs were prescribed to different patients based on underlying characteristics making confounders. It also lacks information about HLA-Cw6 heterozygosis. However, it represents an accurate picture of what occurs in clinical practice. Moreover, our study considered all classes of biologics currently available for treating psoriasis and data were analyzed at week 16 and 48 as in few other studies. However, more data need to be gathered to draw a definite conclusion.

## 5. Conclusions

HLA-Cw6 is known to be one of the most strongly associated psoriasis susceptibility alleles; it is observed to impact the disease course, phenotypic features, severity, comorbidities, and treatment outcomes [6]. HLA-Cw6 status seems to affect the outcomes of different biologics, but long-term data are scant. Our results show long-lasting response to antiTNFα, antiIL17, and antiIL12/23 in HLA-Cw6-positive patients. Of course, more studies are needed to elucidate the role of HLA-Cw6 as a predictor for achievement and maintenance of clinical response to all biologic treatments available. If it was confirmed that the HLA-Cw6 test could well deserve to be integrated into the clinical laboratory work-up.

## Figures and Tables

**Table 1:** Baseline characteristics.

		Overall, *n* = 101	CW6 Positive, *n* = 23	CW6 Negative, *n* = 78	*P*
Age, Mean (sd)		54.80 (13.53)	51.52 (11.46)	55.80 (14.03)	0.186
Disease duration, Median [IQR]		17.00 [13.00, 20.00]	17.00 [17.00, 20.50]	17.00 [12.00, 20.00]	0.234
Gender male, N (%)		70 (70.0)	15 (65.2)	55 (71.4)	0.756
PASI at baseline, Median [IQR]		12.00 [10.00, 15.00]	13.00 [10.00, 15.00]	12.00 [10.00, 15.00]	0.251
HLAB27 positive, N (%)		4 (4.0)	1 (1.0)	3 (3.0)	0.999
Bio-naive, N (%)		76 (75.2)	15 (65.2)	61 (78.2)	0.321
Multi-switch, N (%)		10 (9.9)	3 (13.0)	7 (9.0)	0.860
Subjects with comorbidity, N (%)		67 (66.3)	16 (69.6)	51 (65.4)	0.903
Comorbidities, Median [IQR] {Mean}		1 [0 -1] {0.79}	1 [0 -1] {0.74}	1 [0 -1] {0.81}	0.870
Therapy	antiTNF ^i^, N (%)	41 (40.6)	12 (52.2)	29 (37.2)	0.386
	antiIL17 ^ii^, N (%)	37 (36.6)	6 (26.1)	31 (39.7)	
	antiIL12/23 ^iii^, N (%)	23 (22.8)	5 (21.7)	18 (23.1)	

^i^ Either adalimumab (*n* = 16) or etanercept (*n* = 13) or infliximab (*n* = 9) or certolizumab (*n* = 2) or golimumab (*n* = 1); ^ii^ either secukinumab (*n* = 33) or ixekizumab (*n* = 4); ^iii^ ustekinumab.

**Table 2:** Patients achieving PASI90 at week 16 and week 48.

**Therapy**	**Patients with PASI90 at Week 16**			**Patients with PASI90 at Week 48**		
	**CW6+,** ***n*** **= 23**	**CW6-,** ***n*** **= 78**	***p*** **for Treatment-CW6 Interaction**	**CW6+,** ***n*** **= 23**	**CW6-,** ***n*** **= 78**	***p*** **for Treatment-CW6 Interaction**
Overall (*n* = 101) ^i^, *n* (%)	22 (96)	73 (94)		17 (74)	58 (74)	
antiTNF (*n* = 41) ^i^, *n* (%)	11 (92)	27 (93)	0.610	6 (50)	20 (69)	0.050
antiIL17 (*n* = 37) ^i^, *n* (%)	6 (100)	28 (90)		6 (100)	22 (69)	
antiIL12/23 (*n* = 23) ^i^, *n* (%)	5 (100)	18 (100)		5 (100)	16 (89)	

^i^ All patients were still included in the analysis at week 16 and week 48.
